# The Recombination Triplet State in the Far-Red Light Adapted Photosystem II Is Located at the Chl_D1_ Site and Resides on the Red-Most Chlorophyll of the Reaction Center

**DOI:** 10.1021/acs.jpclett.5c03230

**Published:** 2025-12-12

**Authors:** Andrea Calcinoni, Anna Paola Casazza, Stefania Viola, Alessandro Agostini, Donatella Carbonera, Stefano Santabarbara

**Affiliations:** † Department of Chemical Sciences, University of Padova, Via Marzolo 1, 35131 Padova, Italy; ‡ Photosynthesis Research Unit, 9327 Consiglio Nazionale delle Ricerche, Via A. Corti 12, 20133, Milano, Italy; § Aix Marseille University, CEA, CNRS, BIAM, UMR7265, Saint-Paul-Lez-Durance 13115, France

## Abstract

The energetic limits of Photosystem II (PSII) photochemical reactivity required reconsideration after the discovery of far-red light acclimation responses in cyanobacteria. Insights into PSII functionality following the inclusion of the red-shifted Chlorophylls *d* and *f* can be obtained by extending the current knowledge on spectroscopic and structural properties of its reaction center (RC). The photoinduced triplet states, which represent selective endogenous probes, were therefore investigated in far-red adapted PSII by magnetic resonance techniques. Zero-field splitting tensor analysis combined with spin-polarization dynamics arising from radical pair recombination unambiguously identifies an intrinsically low-energy-absorbing chlorophyll participating in charge separation reactions. The triplet-*minus*-singlet (T–S) spectrum associated with the recombination triplet state, obtained by microwave selection, showed a sharp 725 nm bleaching demonstrating the dominant involvement of this red-shifted chlorophyll in the lowest RC exciton. Moreover, spectral simulations provided strong evidence in favor of its localization at the Chl_D1_ position, making it the most likely site of primary photochemistry.

## Graphical abstract

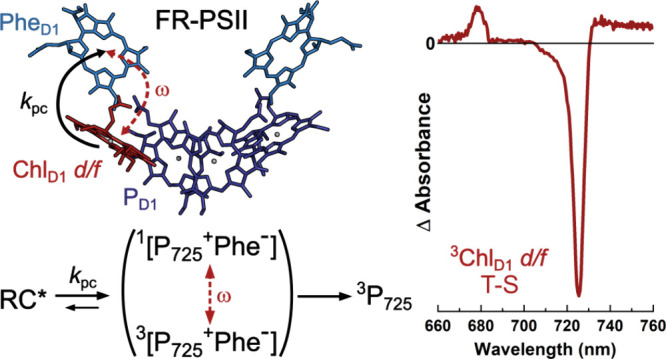

Photosystem II (PSII) is a multisubunit cofactor-binding complex that represents a key component of oxygenic photosynthesis, catalyzing the light-dependent water splitting. The so-called core complex of PSII comprises four main chromophore-cofactor binding subunits: CP43 and CP47 serve as proximal light-harvesting antennae to the photocatalytic reaction center (RC), whose cofactors are primarily coordinated by the D1/D2 heterodimer. In the vast majority of oxygenic phototrophs, Chlorophyll (Chl) *a* is the dominant pigment bound to the core complex, being active both in light harvesting and in photochemistry within the RC, in conjunction with Pheophytin (Pheo) *a* (Figure 1A). The nearly ubiquitous presence of Chl *a* in the RC of PSII has been linked to minimal energy requirements necessary for water splitting, in particular, to the energy of its lowest singlet excited state that lies at approximately ∼ 1.8 eV with respect to the singlet ground state. This general view has come under scrutiny following the discovery of oxygenic phototrophs that contain lower energy absorbing Chls, such as Chl *d*, that replaces almost entirely and constitutively Chl *a* in *Acaryochloris marina*, and Chl *f*, that can replace about 10% of Chl *a* in cyanobacteria capable of the conditional Far-Red Light Photoacclimation (FaRLiP).^1,2^ The FaRLiP response entails a significant reshaping of the photosynthetic apparatus, leading to the replacement of some key subunits of PSI and PSII with specific isoforms.^3^ It has been suggested that, in the far-red (FR) adapted PSII, together with the substitution of some Chl *a* molecules with Chl *f* in CP43 and CP47 to tune the light-harvesting bandwidth, at least one of the Chl *a* participating in photochemical reactions within the RC is replaced by either Chl *f* or Chl *d*.^4^ Investigations by steady-state and time-resolved optical spectroscopy^5–8^ also support the involvement of Chl *d/f* in PSII photochemical charge separation, with early modeling proposing them to be located either at the P_D1/D2_ dimer or at the so-called Chl_D1/D2_ sites.^5^ The assignment of Chl_D1_ being Chl *d* has been favored by Cryo-EM structural studies^9^ and recent QM/MM investigations.^10,11^ Although the assignments are also supported by indirect evidence, such as the identification of possible isoform-specific residues coordinating peripheral substituents of the Chl *d/f* chlorin macrocycles, the unequivocal identification of Chl species remains challenging when the structural coordinates are far from atomic resolution. Hence, the location and molecular identity of the Chl *d/f* molecules in the FR-PSII RC and, consequently, the exact sites of photochemical and charge stabilization events, remain to be proven experimentally. Useful insights to address these issues can be obtained from the analysis of photoinduced triplet states. These species, which are sensitive and selective internal probes of the RC chromophores,^12–14^ were here investigated by both Time-Resolved Electron Paramagnetic Resonance (TR-EPR) and Optically Detected Magnetic Resonance (ODMR) in the FR-PSII. Under reducing conditions, a triplet state displaying the characteristic electron spin polarization (esp) resulting from the radical-pair recombination mechanism was detected. The radical-pair nature of the precursor demonstrates the direct involvement of the triplet-carrying cofactor in the photochemical reactions. The zero-field splitting (ZFS) parameters associated with this triplet state unambiguously demonstrate its localization on either a Chl *d* or a Chl *f* rather than Chl *a* cofactor. Moreover, this chromophore dominates the lowest energy state of the FR-adapted PSII RC, as evidenced by the maximal ground state bleaching at 725 nm of the microwave-induced triplet-*minus*-singlet (T–S) spectrum (Figure 1B), which is more than 40 nm red-shifted (0.11 eV lower energy) relative to the canonical Chl *a*-binding PSII. Spectral simulations assign this low-energy pigment to the Chl_D1_ site. Since the observed lowest energy triplet esp stems from a singlet state radical-pair precursor, it can be concluded that Chl *d/f* at Chl_D1_ is directly involved in primary photochemistry.

**Figure 1:**
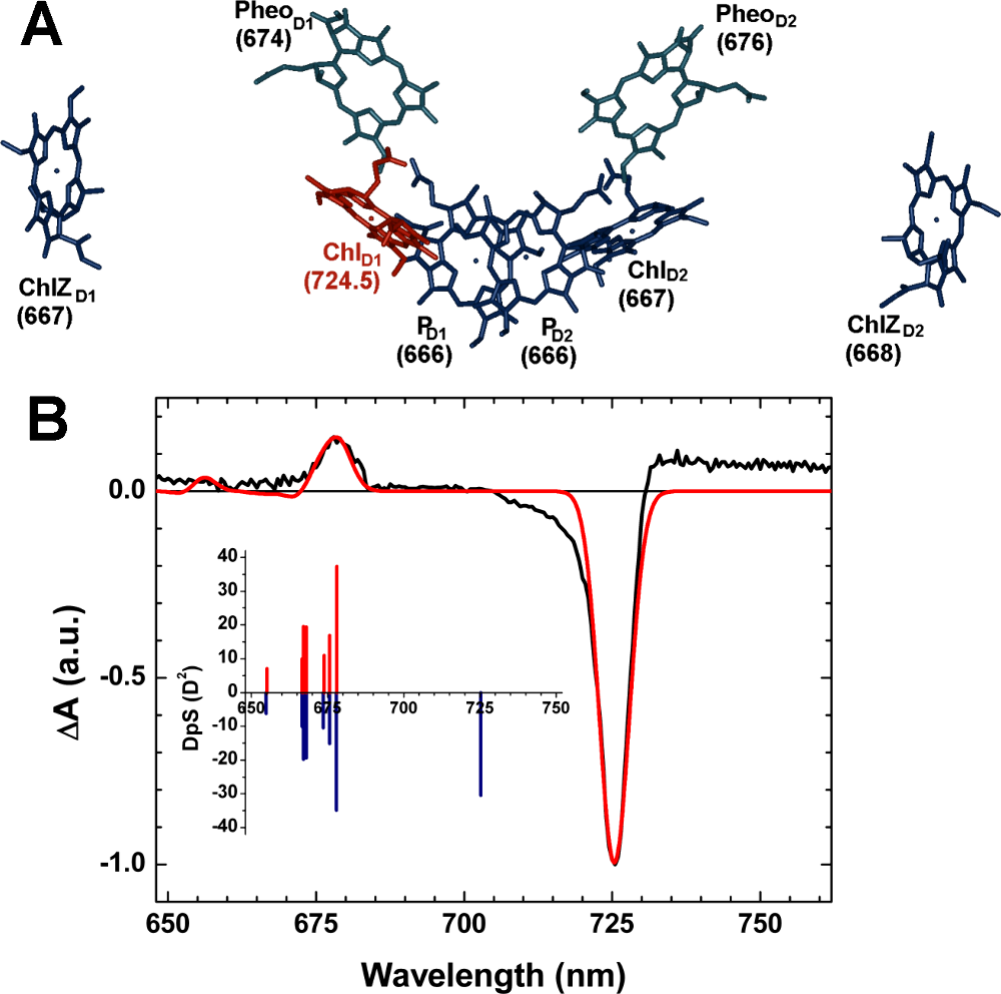
**A:** Chromophore arrangement in the FR-PSII RC (PDB 8EQM) and site energies (in nm) retrieved from T–S spectral simulations considering the photoinduced excited triplet state. **B:** Comparison of the experimental T–S spectrum (black line) acquired upon microwave selection at 570 MHz (marked with a black arrow in Figure 2A) and the simulated one (red line), each normalized to the maximal bleaching. Inset: stick spectra displaying the eigenstates and associated populations for the system being in either the singlet (blue) or triplet (red) state. Further details on the spectral simulation are reported in the Supporting Information.

Measurements were performed on the PSII core complex isolated from thylakoid membranes of *Chroococcidiopsis thermalis* PCC7203 grown under far-red illumination (750 nm; see Supporting Information for further detail). The FR-PSII sample was incubated with Na_2_S_2_O_4_ (10 mM) under anaerobic conditions and illuminated at room temperature in order to fully reduce the PSII quinone acceptors, a condition that, in canonical PSII RC, promotes the recombination of charge-separated states to the triplet state. ODMR analysis on photoinduced FR-PSII triplets was initially conducted by Absorption Detected Magnetic Resonance (ADMR), taking advantage of the differences in the absorption maxima of Chl *a* and Chl *d/f*.

Figure 2A,B shows the ^3^Chl *d/f* ADMR spectra detected at 725 nm, displaying maxima at 566 MHz in the |D| – |E| transition and at 875 MHz, comprising a distinct shoulder at 890 MHz, in the |D| + |E| transition. The marked asymmetry of the |D| + |E| transition suggests the presence of two subpopulations. Gaussian deconvolution of both transitions indicates that the two triplet states have the following ZFS: |D| = 0.0239 cm^–1^, |E| = 0.0052 cm^–1^ and |D| = 0.0244 cm^–1^, |E| = 0.0053 cm^–1^. These correspond to a decrease in |D| and parallel increase in the |E| values with respect to the ZFS previously determined for ^3^Chl *d* either *in vitro* ^15^ or bound to the PSI supercomplex of *A. marina*.^16,17^ At the same time, the ZFS are not largely different from those attributed to a ^3^Chl *f* observed in the recombinant Chlorophyll *f* synthase (rChlF) enzyme (|D| = 0.0251 cm^–1^, |E| = 0.0051 cm^–1^).^18^ Nevertheless, the differences just discussed appear to fall within the ZFS tuning range that protein coordination can exert, as already demonstrated for the thoroughly investigated ^3^Chl *a*.^12,13^ Then, at present, it is not possible to unambiguously assign the observed triplet to either Chl *d* or Chl *f*, whereas the involvement of ^3^Chl *a* can be excluded.

**Figure 2:**
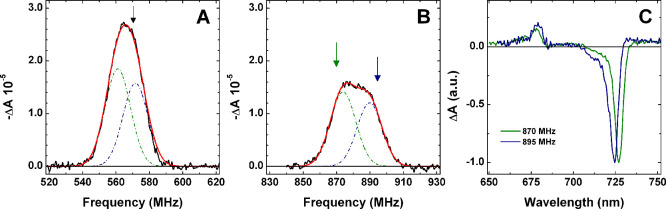
**A** and **B:** ADMR spectra recorded at 725 nm in the ^3^Chl *d/f* |D| – |E| and |D| + |E| transitions, respectively (black lines), together with their decomposition (red lines) by a linear combination of two Gaussian sub-bands (dashed dotted green and blue lines). **C:** T–S spectra recorded upon microwave selection at 870 MHz (green line) and 895 MHz (blue line), preferential for the triplet populations indicated by the arrows in **B**. The T–S spectra are normalized to their maximal bleaching. Experimental conditions: *T* = 1.8 K; Modulation Frequency = 33 Hz; mw power = 0.5 W.

Consistently, the T–S spectrum obtained upon microwave selection at 570 MHz (|D| – |E|) displays a sharp ground state bleaching at 725.5 nm, accompanied by a relatively small amplitude positive absorption feature in the 670 – 685 nm window (Figure 1B). Figure 2C also reports the T–S spectra recorded for preferential selection of the two Chl *d/f* triplet populations discernible in the |D| + |E| transitions, with pump frequencies at 870 and 895 MHz. These T–S spectra show an overall shape similar to the one obtained at 570 MHz, but the exact position of the main bleaching shifts to 727 nm (for 870 MHz) and 724.5 nm (for 895 MHz). The observation of two Chl *d/f* triplet populations having very similar T–S spectra and ZFS parameters, discernible only due to the very high sensitivity of ODMR detection, has been previously reported for the RC triplet state of canonical Chl *a*-binding PSI, ^3^P_700_,^19,20^ where it was attributed to microscopic heterogeneity in pigment–protein coordination of triplet-carrying chromophores. Although a mechanistic assignment of this heterogeneity has not been obtained yet, it could be argued, by analogy, that the two ^3^Chl *d/f* subpopulations observed in FR-PSII arise from similar site-specific conformational, rather than chromophore occupancy, heterogeneity.

It is moreover worth noticing that the double-component heterogeneity of the ^3^Chl *d/f* observed in isolated FR-PSII can also be detected in thylakoids preilluminated under reducing conditions (), where remarkably similar ADMR and T–S spectra are recorded, thereby excluding that it may originate from purification artifacts.

ADMR detection at 682 nm (Figure 3A,B) shows the presence of an additional triplet residing on a Chl *a* molecule, having maxima at 730.5 and 994 MHz, corresponding to ZFS of |D| = 0.0288 cm^–1^, |E| = 0.0044 cm^–1^, which are similar to those reported for canonical Chl *a*-binding PSII under analogous experimental and pretreatment conditions.^21^ Figure 3C shows the T–S spectrum recorded upon microwave selection at 998 MHz (|D| + |E|), which displays a main bleaching at 681 nm and an accompanying positive structure between 670 and 677 nm. The presence of both ^3^Chl *d/f* and ^3^Chl *a* in FR-PSII was also confirmed by FDMR (Fluorescence Detected Magnetic Resonance), under conditions of unselective excitation and broadband fluorescence detection at λ > 715 nm ().

**Figure 3:**
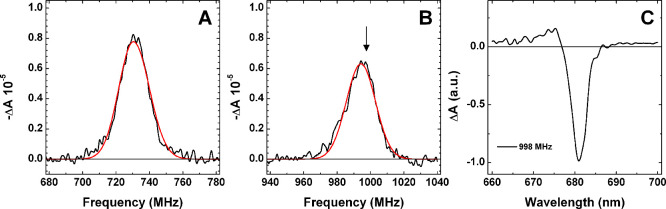
**A** and **B:** ADMR spectra recorded at 682 nm in the ^3^Chl *a* |D| – |E| and |D| + |E| transitions, respectively (black lines), together with their fit by a single Gaussian band (red line). **C:** T–S spectrum obtained upon microwave selection at 998 MHz (indicated by the arrow in **B**), normalized to its maximal bleaching. Experimental conditions are as in Figure 2.

Further insight into the origin of the observed triplet states can be inferred from the population mechanism-dependent esp, which is determinable by TR-EPR. The early time (1.2 – 1.4 μs) TR-EPR spectrum at X-band of FR-PSII under the same reducing conditions employed for the ODMR experiments (Figure 4) confirms the presence of different triplet species. A satisfactorily spectral description is obtained only when considering three triplet components. One is attributed to a carotenoid triplet state that has the broader spectrum and the smallest amplitude. The other two are attributed to ^3^Chl *a* and ^3^Chl *d/f*, based on the respective ZFS parameters (reported together with all simulation parameters and with further detail on the analysis in the Supporting Information). Whereas the ^3^Chl *a* is characterized by an *eee/aaa* esp pattern, indicative of its population by intersystem crossing (ISC), the ^3^Chl *d/f* displays an *aee/aae* polarization, characteristic of population by the radical pair recombination mechanism.^14^

**Figure 4:**
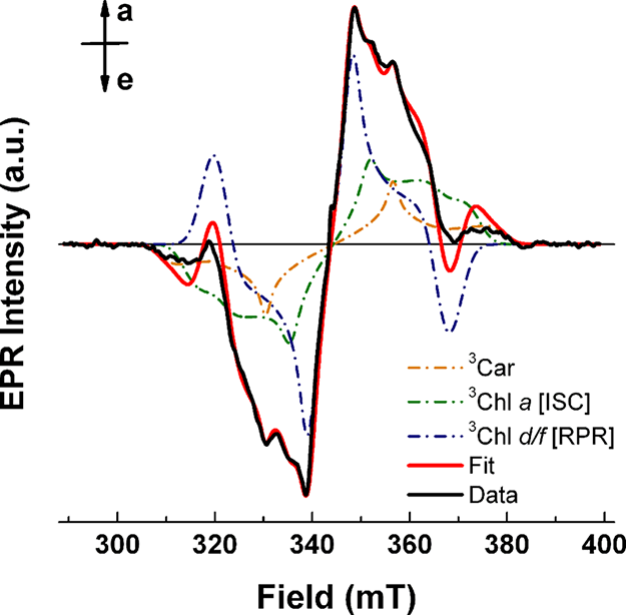
X-band TR-EPR spectrum integrated between 1.2 and 1.4 μs (black line), together with its simulation (red line) resulting from the contributions of a carotenoid triplet state (orange dash-dotted line), a ^3^Chl *a* with intersystem crossing (ISC) esp (green dash-dotted line), and a ^3^Chl *d/f* with a radical pair recombination (RPR) esp (blue dash-dotted line). Experimental conditions: *T* = 80 K; υ= 9.65 GHz; mw power: 0.6 mW; excitation wavelength: 532 nm.

## On the Origin of the Observed Chlorophyll a Triplet State

The analysis of both ODMR spectra (Figures 3 and ) and TR-EPR (Figure 4) demonstrates the presence of a photoinduced ^3^Chl *a* triplet in the FR-PSII core complex preparation. Similar signals could also be recorded by ADMR of thylakoid membranes from FR-acclimated cells (). Hence, the observed ^3^Chl *a* does not appear to be due to possible purification artifacts, since thylakoids are a rather intact environment.

It is worth noting that the T–S spectrum associated with the ^3^Chl *a* triplet in FR-PSII closely resembles that of P_680_ recombination triplet in the canonical PSII reaction center.^21^ Thus, the simplest explanation would be that the observed ^3^Chl *a* resides in a residual population of PSII complexes that has not undergone the FR acclimation and is therefore, substantially, a canonical Chl *a*-binding PSII. A fundamental point that argues against this interpretation is that a typical Chl *a*-binding PSII is expected to give rise, under the experimental conditions employed in this study, to a triplet populated by the radical pair mechanism, whereas the TR-EPR spectrum analysis indicates the population by ISC (Figure 4). A different explanation for the observed signal shall then be sought. A proposition that would accommodate the just discussed ^3^Chl *a* characteristics is that of considering its formation in a subpopulation of PSII core-like complex harboring the intrinsic red-shifted Chls *d*/*f* in CP43 and CP47, but only Chl *a* in the RC (or D1/D2 complex). The ISC mechanism in this RC subpopulation would then be the result of a perturbation of the cofactor energetics, for instance, resulting from the specific amino acid substitution present in the far-red isoforms, that would tend to favor ISC with respect to radical pair recombination, similarly to the situation already discussed for the PSI reaction center of *Acaryochloris marina*.^16,17^

It is interesting to notice that the characteristics of the ^3^Chl *a* detected in this study show some significant resemblance to those previously reported for the recombinant Chl *f* synthase (rChlF) from *Fischerella thermalis* where a ^3^Chl *a*, populated by the ISC mechanism while being characterized by an ODMR-detected T–S spectrum with a maximal bleaching at ∼ 680 nm and an overall shape similar to that of ^3^P_680_, was observed.^18^ Moreover, the ZFS and the sublevel triplet populations of the ^3^Car detected in TR-EPR experiments (Figure 4) are also closely comparable to those retrieved in the rChlF,^18^ suggesting an analogous assignment. However, the rChlF analyzed in the mentioned study was a homodimer of the so-called rogue psbA4 D1 isoform,^22^ lacking the antenna complement. The substantial copurification of the pigment–protein complexes carrying this ^3^Chl *a* with the FR-PSII suggests a core-like form of the native ChlF, instead. Therefore, a scenario in which the rogue psbA4 D1 isoform is part of a modified core-like antenna-harboring structure is here proposed, similarly to the assembly already discussed by Trinugroho et al.^23^ This suggestion certainly requires further experimental support, but remarkably, it would imply the constitutive presence of ChlF in thylakoids and cells under conditions promoting the FaRLiP response.

## The Chl d/f Triplet Is Localized in the FR-PSII Reaction Center

At variance with the uncertainties about the origin of the identified ^3^Chl *a*, the population mechanism of the ^3^Chl *d/f* involving charge recombination from a radical pair precursor links this species directly to photochemical reactions taking place in the RC of FR-PSII. To assess the specific binding position of Chl *d/f*, generally considered a pivotal matter of discussion in far-red light photosystems, simulations of the T–S spectrum in Figure 1B were performed via diagonalization of an excitonic Hamiltonian (theoretical details are reported in Supporting Information). Couplings between chromophores were computed under the point-dipole approximation, taking the geometrical arrangement from either a canonical PSII (PDB 3WU2 ^24^) or from a FR-PSII (PDB 8EQM^9^). Since site energies of FR-PSII RC are still relatively undefined, calculations were initially executed using the ones previously retrieved for the Chl *a*-binding PSII RC^25^ (as reported in parentheses in Figure 1A), except for the Chl *d/f* site energy, which was tuned to match the experimental bleaching at 725.5 nm. The most satisfactory simulation favors the localization of the ^3^Chl *d/f* on the cofactor occupying the Chl_D1_ position in the RC (Figure 1B). An adequate description of the T–S spectrum can also be obtained by placing the low-energy Chl *d/f* chromophore at the Chl_D2_ position (), which is, however, part of the electron transfer inactive branch, making this location inconsistent with the detected triplet population by radical pair recombination. Locating the triplet-carrying Chl *d/f* at either the P_D1_ or P_D2_ sites largely worsened the spectral simulation (). Further, considering the possible presence of more than one Chl *d/f* molecule in the RC (for example, in both Chl_D1_ and Chl_D2_) did not lead to any significant improvement of the simulations either (). Because of the limited information concerning the site energies of RC chromophores of FR-PSII, simulations were also performed considering a hypothetical simplified scenario, where all non-Chl *d/f* chromophores were taken as being isoenergetic at 666 nm. Also in this case, the closest agreement between the simulations and experimental T–S spectra was obtained by localizing the triplet on a low-energy state at the Chl_D1_ position (). These results indicate that the excitonic calculations performed within the point-dipole approximation are not very dependent on the specific site energies adopted for the Chl *a*/Pheo *a* cofactors but are specifically sensitive to the location of the single far-red absorbing pigment instead. This in turn allows a reliable determination of the low-energy state within the FR-PSII RC, which represents a significant piece of information considering also the current debate on this matter.

The assignment of the low-energy ^3^Chl *d/f* to the Chl_D1_ chromophore is consistent, and hence confirms, the conclusions of previous studies that identified it as the most probable locus for a red-shifted chlorophyll in FR-adapted PSII RC.^4,8–11^ The Chl_D1_ site is generally considered to be the most likely location of the recombination triplet also in canonical Chl *a*-binding PSII RC,^26–28^ at least at cryogenic temperatures, as well as the lowest-energy site in the RC^*e*.*g*.^.^29–35^ The main difference between the canonical and the FR-PSII RC is then the excited state energy of the chromophore occupying the Chl_D1_ site. In the case of Chl *d/f*-binding PSII, the Chl_D1_ excited state has a significantly lower energy (∼100 meV) than the other RC eigenstates, whereas in Chl *a*- binding PSII RC a more contained energy spread occurs.

The relatively small energy differences in canonical PSII will lead to a temperature-dependent localization of the singlet excited state on the moderately red-shifted, lowest energy site of the RC, with the excited state being already partially delocalized at low temperatures^*e*.*g*.^ ^32,34^ and expectedly even more at room temperature, although the stronger charge transfer character of the Chl_D1_Pheo_D1_ pair might promote to some extent the localization of charge separation has also been suggested^*e*.*g*.^.^33,35^ In the case of FR-PSII, however, excited-state localization on the further red-shifted site occupied by the Chl *d/f* molecule will be strong already at higher, physiological temperatures, thanks to its considerable lower energy. As a result, in FR-PSII, Chl_D1_ not only represents the lowest-energy RC state but is also the energetically preferred site for primary photochemical charge separation and recombination, irrespective of temperature.

Low-energy states in the FR-PSII RC are essential to ensure sufficient RC excited-state population under FR illumination, thereby compensating for the simultaneous presence of Chl *d/f* in the CP43 and CP47 antenna complexes. Furthermore, the presence of a *single*, well-defined low-energy state in the RC concentrates the excitation on the specific site most likely to initiate photochemistry, giving rise to the [*P* ^+^_725_ *Phe* ^–^_D1_] charge separated state, where *P* ^(+)^_725_ is the Chl *d/f* molecule at the structural Chl_D1_ site. Electron transfer would then proceed through hole transfer, leading to the formation of the secondary radical pair [*P* ^+^_*D1(/D2)*_ *Phe* ^–^_*D1*_], where *P* ^+^ *_D1(/D2)_* are Chl *a* molecules at the respective structural sites. The most likely mechanism of triplet population, under reducing conditions, involves the recombination from the primary radical pair, according to the following kinetic scheme: $RC^{*}\;\overset{k_{pc}}{⇄}$ ([P725+PheD1−]1 ↔ω[P725+PheD1−]3) →P7253 , where *k*_*pc*_ is the rate of primary photochemistry, and ω is the rate of singlet-triple mixing between the singlet and the triplet state of the [*P* ^+^_725_ *Phe* ^–^_D1_] spin-correlated radical pair, finally leading to triplet localization on *P*_725_. This reaction scheme implies that singlet–triplet mixing kinetically outcompetes the population of the secondary radical pair or that, alternatively, this reaction is inhibited under the measuring conditions employed. Possible triplet population mechanisms involving the recombination of a secondary radical pair are discussed in the Supporting Information.

In conclusion, the RC asymmetry brought about by the incorporation of a single Chl *d/f* molecule and the resulting enhanced excited-state localization on the putative primary donor, which is expected to be already significant at physiologically relevant temperatures, represents a crucial bioenergetic reorganization that preserves both the quantum conversion efficiency of FR-PSII and the water-splitting activity in view of the substantial conservation of the donor-side electron transfer cofactors, P_D1_ and Tyr Z.

## Supplementary Material





## Notes

Additional detail concerning biochemical and spectroscopic methodologies, data analysis and spectral simulations. ODMR studies on thylakoid membrane and their analysis. A discussion of the different pathways leading to the population of the recombination triplet population (PDF)
Transparent Peer Review report available (PDF)

## Abbreviations

A/FDMR: Absorption/Fluorescence Detected Magnetic Resonance
Chl: Chlorophyll
(r)­ChlF: (recombinant) Chlorophyll *f* synthase
esp: electron spin polarization
FR: Far Red
ISC: Intersystem Crossing
ODMR: Optically Detected Magnetic Resonance
PSII: Photosystem II
RC: Reaction Center
TR-EPR: Time-Resolved Electron Paramagnetic Resonance
ZFS: Zero Field Splitting
