# Supplementary material for: The Recombination Triplet State in the Far-Red Light Adapted Photosystem II Is Located at the ChlD1 Site and Resides on the Red-Most Chlorophyll of the Reaction Center
Source: J Phys Chem Lett. 2025 Dec 12;16(51):13238–43. doi: 10.1021/acs.jpclett.5c03230 (PMC12746436; doi:10.1021/acs.jpclett.5c03230)
Supplement: Supplementary file 2 [file jz5c03230_si_002.pdf]

jz-2025-03230t.R1

Name: Peer Review Information for "The Recombination Triplet State in the Far-Red Light Adapted Photosystem II Is Located at the ChlD1 Site and Resides on the Red-Most Chlorophyll of the Reaction Center"

First Round of Reviewer Comments

Reviewer: 1

Comments to the Author

This is a very nice piece of work showing the formation a charge recombination triplet state on a red chlorophyll in far-red light adapted PSII. This provides unambiguous evidence for the presence of Chl d or Chl f in the core of PSII under these conditions.

Based on simulations of the singlet minus triplet spectra the authors propose that the red chlorophyll is located in the D1 site.

They should consider the following before the paper is published. Perhaps I missed it but I don't think they referenced the original work from Rutherford and co-workers from the 1990's in which they used partially oriented samples to show that the charge recombination triplet resides on the accessory chlorophyll Chl D1 in white light adapted PSII. Thus, the location of the recombination triplet on ChlD1 is not unique red-light adapted PSII and apparently does not depend on the type of chlorophyll molecule in this site.

This authors should mention this and discuss the different possible models for the initial charge separation and recombination, including the possibility that the triplet is formed first on P680 and is then transferred to Chl D1.

Reviewer: 2

Comments to the Author

The manuscript by Calcinoni et al. presents magnetic resonance studies on the reaction center of cyanobacterial Photosystem II. Specifically the study focuses on the characterization and attribution of triplet states in far-red PSII, which can contain red-shifted chlorophyll variants (Chl d or f, instead of Chl a) in the reaction center.

An important question in this subfield of photosynthesis research historically concerned the exact location of this red-shifted chlorophyll among the four chlorophylls of the PSII RC. At this point in time there is strong support from multiple lines of evidence (Cryo-EM structures, modeling, spectroscopy, mutation studies, etc) that Chl d/f occupies the site known as ChlD1.

The present study examines photo-induced triplet states in the RC using time-resolved EPR and optically detected magnetic resonance. At least three triplet states can be discerned from the data, and they are attributed to chemically distinct pigments. Interpretation of zero field splitting parameters lead to the conclusion that one of the triplet states is localized on a Chl d/f rather than Chl a pigment, another on a Chl a pigment, and another is tentatively attributed to a carotenoid. The Chl involved dominates the low-energy state of far-red PSII, and the authors suggest it should be assigned to the ChlD1 site. The mechanism of creation of this triplet state (radical pair recombination instead of intersystem crossing) is consistent with participation in primary photochemistry.

Although I acknowledge the amount of effort and the complexity of the work presented in this manuscript, I think there are limitations and weaknesses that do not support publication of the manuscript as a Letter. The work can be considered confirmatory of existing assignments regarding cofactor identities, but even so the conclusion regarding cofactor assignment does not uniquely follow from the data. Therefore, I see more value in better documenting the experimental work and explaining the analysis rather than in physical insights.

- I find a lot of the material contained in the SI, particularly sections 3-7, to contain essential information both in terms of data and in terms of interpretation. These should be fully shown in a full paper, otherwise significant arguments (but also some ambiguities in the data and limitations in interpretation) are not properly shown.

- The simulations (Figure S6) cannot really differentiate between D1 and D2 sites.

- Discussion in the manuscript suggests that the FR-PSII is different from the Chl a-only PSII in essential ways. But this contradicts both older and recent research in advanced

spectroscopy (Fleming, Ogilvie) and theory (Sirohiwal, Pantazis) which supports localization rather than delocalization of excitons in the RC and points to ChlD1 as a clearly differentiated red-shifted Chl a, and the site of the lowest energy triplet also in Chl a-only PSII.

- No sufficient rationalization exists for the presence of two triplet state populations attributable to Chl d/f.

- The triplet Chl a is an important component that remains unassigned. The discussion in the SI should be transferred to the main text.

- The excitonic calculations are not sensible because an isoenergetic system (all Chl a and Pheo a site energies set at 666 nm) by definition cannot function as a reaction center. The fact that it is hard to see significant differences anyway (Figure S7), suggests that there is not much information to be extracted from such simulations.

Author's Response to Peer Review Comments:

Answers to the reviewers' comments:

Reviewer 1

Comments: This is a very nice piece of work showing the formation a charge recombination triplet state on a red chlorophyll in far-red light adapted PSII. This provides unambiguous evidence for the presence of Chl d or Chl f in the core of PSII under these conditions. Based on simulations of the singlet minus triplet spectra the authors propose that the red chlorophyll is located in the D1 site. They should consider the following before the paper is published. Perhaps I missed it but I don't think they referenced the original work from Rutherford and co-workers from the 1990's in which they used partially oriented samples to show that the charge recombination triplet resides on the accessory chlorophyll Chl D1 in white light adapted PSII. Thus, the location of the recombination triplet on ChlD1 is not unique red-light adapted PSII and apparently does not depend on the type of chlorophyll molecule in this site.

Answer: We thank the reviewer for the positive assessment of the manuscript and for the useful suggestions. The discussion concerning the localisation of the recombination triplet in far-red light adapted PSII has now been modified and, we trust, improved, to avoid what appear to have been a misunderstanding concerning the difference with canonical Chl a-

only-binding reaction centre. We fully agree with the reviewer comments that the original suggestion for the recombination triplet in the canonical RC on what is now known as the ChlD1 site comes from EPR studies on oriented particles by Rutherford and co-workers, and at low temperature confirmed by other investigators over the years. Hence, as indicated by the reviewer, the triplet localisation in canonical and FR-light adapted PSII would be the same. The difference resides on the larger energy difference in site energy brought about by the insertion of an intrinsically low-energy pigment, with respect to moderately low-energy Chl a form in the canonical PSII RC.

Comments: This authors should mention this and discuss the different possible models for the initial charge separation and recombination, including the possibility that the triplet is formed first on P680 and is then transferred to Chl D1.

Answer: We thank the reviewer for the suggestion. However, we have decided to prefer omitting in the main text more complex recombination scenarios than the one originating from the primary radical pair for simplicity, and also because we considered them to occur less likely. The proposed population from the secondary radical pair can not be in principle ruled out, but it would most likely involve triplet-triplet energy transfer from the triplet formed on the Chl a composing PD1(/D2) molecules to the Chl d/f at the ChlD1 site. However, triplet-triplet transfer is generally fairly demanding and requires rather strong coupling for being efficient, which makes it, in our opinion, less likely than the population directly from the primary radical pair. Note that, for the observed recombination spin polarisation patter (shown in the TR-EPR experiment) to be conserved, a very fast triplet-triplet transfer must occur, avoiding spin relaxations to ms [illegible] 0 states. This is again considered here to be unlikely due to the mentioned strong coupling requirements. Nevertheless, alternative populations mechanisms are included in the final paragraph of the Supporting Information.

Reviewer 2:

Comments: The manuscript by Calcinoni et al. presents magnetic resonance studies on the reaction center of cyanobacterial Photosystem II. Specifically the study focuses on the characterization and attribution of triplet states in far-red PSII, which can contain red-shifted chlorophyll variants (Chl d or f, instead of Chl a) in the reaction center.

An important question in this subfield of photosynthesis research historically concerned the exact location of this red-shifted chlorophyll among the four chlorophylls of the PSII RC. At this point in time there is strong support from multiple lines of evidence (Cryo-EM structures, modeling, spectroscopy, mutation studies, etc) that Chl d/f occupies the site known as ChlD1.

The present study examines photo-induced triplet states in the RC using time-resolved EPR and optically detected magnetic resonance. At least three triplet states can be discerned from the data, and they are attributed to chemically distinct pigments. Interpretation of zero field splitting parameters lead to the conclusion that one of the triplet states is localized on a Chl d/f rather than Chl a pigment, another on a Chl a pigment, and another is tentatively attributed to a carotenoid. The Chl involved dominates the low-energy state of far-red PSII, and the authors suggest it should be assigned to the ChlD1 site. The mechanism of creation of this triplet state (radical pair recombination instead of intersystem crossing) is consistent with participation in primary photochemistry.

Although I acknowledge the amount of effort and the complexity of the work presented in this manuscript, I think there are limitations and weaknesses that do not support publication of the manuscript as a Letter. The work can be considered confirmatory of existing assignments regarding cofactor identities, but even so the conclusion regarding cofactor assignment does not uniquely follow from the data. Therefore, I see more value in better documenting the experimental work and explaining the analysis rather than in physical insights.

Answer: We thank the reviewer for the evaluation of the manuscript, but we think that the manuscript provides indeed sufficient new direct information to be granted publication as a Letter. On the one hand, we agree with the reviewer comment that the localisation of Chl d/f in the PSII reaction centre of far-red adapting organism on the ChlD1 locus has been previously suggested, based on different observations (however we are not aware of any published or reported mutagenesis studies at regard). Most of the spectroscopic investigation reported so far, however, relied on rather indirect assignments, for instance requiring fairly heavy kinetic modelling, or from indirect signals (e.g. local stark-effect/electrochromic, resulting from chromophores photo-accumulation). The here presented data, by combining the determination of ZFS and triplet population mechanism from a radical pair precursor, unambiguously demonstrate the involvement of the intrinsically low-energy Chl d/f in primary photochemical charge separation in this reaction centre. We believe this information, perhaps partially confirmative, still provides an important contribution, unambiguously demonstrating that PSII photochemistry, and as a

result, water splitting, can be driven by energy lower than the energetic limit imposed by the Chl a excited state, a topic that is of general interest, even outside the photosynthetic community.

The answer to specific comments is provided below:

- I find a lot of the material contained in the SI, particularly sections 3-7, to contain essential information both in terms of data and in terms of interpretation. These should be fully shown in a full paper, otherwise significant arguments (but also some ambiguities in the data and limitations in interpretation) are not properly shown.

Answer: In our opinion most of data in the SI are confirmative of what already shown in the main text, but do not add any additional content. The T-S spectra in full scale are not necessary since analysis of the triplet-triplet transitions (beyond 740 nm) are not part of the focus of the presented work. FDMR experiments confirm and support the triplet identified by ADMR in the main text. ADMR and T-S recorded in thylakoids confirm that the signal measured in the isolated photosystem are not artefacts, but do not show additional information. The analysis of TR-EPR reports the same spectra already shown in the main text; the explicative spectra for the different population mechanisms aid in substantiating the quality of the TR-spectral fits/decomposition but, again, add no additional information. The simulations of T-S for the low-energy state in different positions within the RC are discussed in the further comments advanced by the reviewer.

- The simulations (Figure S6) cannot really differentiate between D1 and D2 sites.

Answer: we agree with the reviewer comment; the results concerning the ChlD2 localisation are now further discussed in the main text. Although the T-S can be satisfactorily simulated by placing the Chl d/f molecule at the ChlD2, this represents the "inactive" electron transfer branch of the reaction centre. Hence, the formation of primary radical pair in the inactive branch would be inconsistent with the functionality of the photosystem. It is also extremely unlikely that the normally inactive branch becomes the active one in the far-red adapted PSII RC, because this would require a complete reconfiguration of the functionality of all redox cofactors and especially of the quinone acceptors (Qa and Qb, with Qb becoming the primary acceptor, to accept electron from the Pheo on the D2 cofactor chain). We trust these not to be very feasible, and hence, discard D2 as the site of the Chl d/f localisation, even if the T-S is correctly reproduced.

- Discussion in the manuscript suggests that the FR-PSII is different from the Chl a-only PSII in essential ways. But this contradicts both older and recent research in advanced spectroscopy (Fleming, Ogilvie) and theory (Sirohiwal, Pantazis) which supports

localization rather than delocalization of excitons in the RC and points to ChlD1 as a clearly differentiated red-shifted Chl a, and the site of the lowest energy triplet also in Chl a-only PSII.

Answer: we trust this is a misunderstanding and apologise for the lack of clarity in the way the discussion paragraph was previously sentenced. We agree with the reviewer comment that also in the canonical Chl a-only binding reaction centre of PSII, ChlD1 represents the lowest energy state and the most likely site of recombination triplet localisation, at least at low temperatures. The point we would like to make, and that we trust we have explained by rephrasing the closing discussion paragraphs, is that the insertion of an intrinsically low-energy chromophore further increases the singlet excited localisation on this site not only at low/cryogenic temperatures, that is the condition in which some of the mentioned experimental paper have been performed, but also at room temperature, that is, under physiologically relevant conditions. In the case of canonical PSII RC, population would be already significantly, but not entirely, localised on this chromophore at RT.

- No sufficient rationalization exists for the presence of two triplet state populations attributable to Chl d/f.

Answer: this issue has now been further discussed in the main text. The observation of two triplet sub-populations has been already reported in several ODMR investigations of (Chl a-binding) Photosystem I (Carbonera et al. 1997 doi: 10.1016/S0005-2728(97)00068-6, and is also observable for the photosystem embedded in thylakoids and even algal cells Santabarbara et al. 2002 doi: 10.1016/j.bbabio.2006.10.007, hence excluding an artefact); unfortunately, a more mechanistic interpretation of this heterogeneity has not been obtained yet, not even in an abundantly studied photosystem. It is nonetheless an interesting observation that this kind of heterogeneity, likely deriving from subtle differences in the chromophore coordination, is here detected in the FR-PSII whereas WL-PSII typically displays well-defined/homogenous ODMR transitions. Although we are not able to offer an explanation, we feel that the experimental data merit to be presented.

- The triplet Chl a is an important component that remains unassigned. The discussion in the SI should be transferred to the main text.

Answer: we followed the reviewer suggestion; the discussion on the origin of the Chl a triplet has now been transferred to the main text.

- The excitonic calculations are not sensible because an isoenergetic system (all Chl a and Pheo a site energies set at 666 nm) by definition cannot function as a reaction center. The

fact that it is hard to see significant differences anyway (Figure S7), suggests that there is not much information to be extracted from such simulations.

Answer: we disagree with this specific comment. Considerations of iso-energetic system are widespread and we think they have been very insightful over the decades. The simulations for a Chl a/Pheo a isoenergetic system indicate that the conclusions reached are not very sensitive to the specific site energy of these chromophores, on this we agree with the reviewer, but are instead sensitive on the triplet localisation, which is the information we aim at extracting from the experimental results. Hence, the results in FigureS7 serve to prove the robustness of the assignment only. A short discussion on the results obtained for the isoenergetic simulations was included in the main text anyways. Assigning accurate site energies to the remaining sites is outside of the scope of the investigation. Noticeably, however, the site energies adopted are taken from the independent computations of Renger and coworkers, which instead aimed at a detailed description of canonical Chl a-only PSII RC, and shall therefore be considered, if not accurate, at least reasonable.

jz-2025-03230t.R2

Name: Peer Review Information for "The Recombination Triplet State in the Far-Red Light Adapted Photosystem II Is Located at the ChlD1 Site and Resides on the Red-Most Chlorophyll of the Reaction Center"

Second Round of Reviewer Comments

Reviewer: 1

Comments to the Author

The revisions made by the authors address the suggestions in my original review and, in my view, the paper is now acceptable for publication.

Author's Response to Peer Review Comments:

We thank the reviewer for the positive assessment of the revised version of our manuscript.

The Supplementary Information document format has been updated to include the specific changes in editing requested by the editorial office.
